# The use of social media platforms by migrant populations during the COVID-19 pandemic

**DOI:** 10.1093/eurpub/ckac130.177

**Published:** 2022-10-25

**Authors:** S Hargreaves, LP Goldsmith, M Rowland-Pomp, K Hanson, A Deal, AF Crawshaw, A Ahmad, M Razai, T Vandrevala

**Affiliations:** 1 Institute for Infection and Immunity, St George's University of London, London, UK; 2 Faculty of Health, Social Care and Education, Kingston University & St George’s, London, UK; 3 Population Health Research Institute, St George's University of London, London, UK

## Abstract

**Background:**

The rapid expansion of internet and social media use has meant that both useful and potentially harmful health information can spread rapidly. Groups experiencing barriers to health systems may be more reliant on social media as a source of health information. We did a systematic review to determine the extent and nature of social media use in migrant and ethnic minority communities for COVID-19 information, and implications for preventative health measures including vaccination intent and uptake.

**Methods:**

We reviewed published and grey literature following PRISMA guidelines (PROSPERO registered CRD42021259190). Global research was included that reported on the use of social media by migrants and/or ethnic minority groups in relation to COVID-19.

**Results:**

1849 unique records were screened, and 21 data sources included in our analysis involving studies from the UK, US, China, Jordan, Qatar, and Turkey. We found evidence of consistent use of a range of social media platforms for COVID-19 information in some migrant and ethnic minority populations (including WeChat, Facebook, WhatsApp, Instagram, Twitter, YouTube), which may stem from difficulty in accessing COVID-19 information in their native languages or from trusted sources. There were positive and negative associations with social media use reported, with some evidence suggesting circulating misinformation and social media use may be associated with lower participation in preventative health measures, including vaccine intent and uptake, findings of which are likely relevant to multiple population groups.

**Conclusions:**

Urgent actions and further research are now needed to better understand the use of social media platforms for accessing health information by groups who may be marginalised from health systems, effective approaches to tackling circulating misinformation, and to seize on opportunities to make better use of social media platforms to support public health communication.

**Key messages:**

- We found evidence of consistent use of a range of social media platforms for COVID-19 information in some migrant and ethnic minority populations.
- Further research is needed to better understand the use of social media platforms for accessing health information by groups who may be marginalised from health systems.

